# Fruit availability for migratory birds: a GIS approach

**DOI:** 10.7717/peerj.6394

**Published:** 2019-02-05

**Authors:** Clara Tattoni, Erica Soardi, Filippo Prosser, Maurizio Odasso, Paolo Zatelli, Marco Ciolli

**Affiliations:** 1 Dipartimento di Ingegneria Civile Ambientale e Meccanica, Università degli Studi di Trento, Trento, Italy; 2 Sezione di Botanica, Fondazione Museo Civico di Rovereto, Rovereto, Italy; 3 PAN Studio Associato, Pergine Valsugana (TN), Italy

**Keywords:** Open Source GIS, Fruiting phenology, Forest types, Wild berries, Bird feeding guilds, Kernel geo-statistics

## Abstract

Bird migration is a widely studied phenomenon, however many factors that influence migratory flows remain unknown or poorly understood. Food availability en route is particularly important for many species and can affect their migration success, pattern and timing but this relationship has not been addressed at a wide scale due to the lack of spatial models of food availability on the terrain. This work presents a GIS-database approach that combines spatial and non-spatial ecological information in order to map fruit availability from vegetation over time in the SE Alps, an important node of European migratory routes. We created a unique database that contains information on the presence and periods of fructification of 52 wild plants carrying berries and a series of original cartographic themes. The presence and coverage of the plant species was modelled with the geo-statistical method of the Gaussian Kernel, which was validated against the ground truth of field sampling data with a correct classification power above 80% in most cases. The highest fruit availability in the study area during September and October co-occurs with the peak of captures of berry eating birds. The maps created and distributed along this work can be useful to address more detailed studies about stopover sites as well as the spatial ecology of other fruit eating animals.

## Introduction

Bird migration is one of the most extraordinary natural phenomena that has always fascinated humans and stimulated researchers curiosity. Although migration is increasingly studied, many factors that influence migratory flows and routes still remain unknown or poorly understood (Bairlein, 2003; Bairlein, 2008). Different works studied migration paths and birds features taking into account various parameters (Chevallier et al., 2010; Vilkov, 2013; Bauer, Ens & Klaassen, 2010). The relation between migration paths and trophic availability en route is particularly difficult to study, since it is difficult to model food availability on the terrain (Drent, Fox & Stahl, 2006; Ma, Li & Chen, 2005; Moore & Woodrey, 1999; Gyimóthy et al., 2011). This is due both to scarce vegetation data availability and to an imprecise knowledge of bird migration paths. The habitats or natural communities that provide migrants with the opportunity to refuel and rest during their journey are commonly known as stopover sites. They can be well delimited spots like a lake or a marsh for water birds (Downs & Horner, 2008) or can be more widely distributed along a gradient of environments like a combination of different forest vegetation types located following the orientation of an alpine valley. Finding food during the migration route is extremely important for many migratory species and can affect their migration success, pattern and timing (Drent, Fox & Stahl, 2006; Newton, 2006; Vilkov, 2013; Wolfe, Johnson & Ralph, 2014).

Although many associate migration with migratory flight, according to Hedenström & Alerstam (1988), most of the migration period is spent stationary at successive stopover sites where birds spend their time resting and foraging as they rebuild protein and energy stores in preparation for their next migratory flight. Also McWilliams et al. (2004), underlining the role of stopover sites for the diet of migratory birds, stressed that the quality of the food available at stopover sites can be dramatically important. Migrant species captured in autumn had significantly greater body masses and greater daily rates of body mass gain at sites where fruits were available compared to those of birds taken at sites without fruits (Ferns, 1975; Thomas, 1979; Bairlein, 2002). Experimental removal of available fruits decreased local abundance of autumn migrants (Parrish, 2000) and birds overwintering (Borgmann et al., 2004). Therefore, seasonally abundant fruits can be a significant food resource for migrating songbirds in temperate regions (Parrish, 1997; White, 1989; Smith et al., 2007). Oguchi, Smith & Owen (2017) affirm that migrating birds can improve their immune and antioxidant status during stopover, implying that variation in stopover habitat can affect migrants’ health and underlining the importance of stopover site ecological conservation.

Several attempts have been performed to study this complicated issue, for example Wade & Hickey (2008) used a combination of satellite imagery processing that were statistically compared with data collected in the field, mapped according to locations of birds’ preferred food in an aquatic environment, with the aim to prioritize research and conservation efforts in these areas. Mudrzynski & Norment (2013) studied the influence of habitat structure and fruit availability on the use of a stopover site by songbirds, with the aim to understand if the successional stages of forest affect birds food’s availability. McClure, Rolek & Hill (2012) investigated the importance of micro-habitat information in predicting occupancy of wintering migratory birds. The need to analyse stopovers at multi-scale level was highlighted by Buler, Moore & Woltmann (2007).

In Europe, birds migrate in autumn from central or northern Europe towards southern African wintering areas (post-nuptial migration) while in spring, the species come back towards reproduction and nesting areas (pre-nuptial migration) (Berthold, 2001). During these periodic mass movements between Europe and Africa, wildfowl must face a long journey encountering threats and obstacles represented by geographic barriers like the Alps, the Mediterranean Sea and Sahara Desert, (the latter for long range migratory birds). Nevertheless, many birds pass through the Alps during autumn migration and researchers are trying to understand the reasons (Bruderer & Jenni, 1988). Trophic availability in the Alpine area seems to play a fundamental role in this migratory route (Bruderer & Jenni, 1990).

Alpine valleys are relatively more natural than the surrounding European plains, that are interested by intensive agriculture and urbanization. Similarly, according to Stach et al. (2016) the migration across the Asian mountains is possibly a response to local food availability, but they did not provide a quantitative estimation of the available energy at stopovers. Ogden, Martin & Williams (2013) highlighted the importance of Alpine and sub-alpine stopovers in fat accumulation of many migratory birds also in American mountain environments. The majority of the birds captured in the Alpine ringing stations show larger fat reserves than their co-specific captured in the plains (Bruderer & Jenni, 1990; Spina, 2011). The seasonal availability of fruits and berries is one of the main resources for those passerines that become mainly frugivorous during migration. Migrant abundance is highest in habitats with greater fruit availability during autumn migration (Martin & Karr, 1986; Blake & Hoppes, 1986; Rodewald & Brittingham, 2004; Suthers, Bickal & Rodewald, 2002) therefore the conservation and management of Alpine stopover areas is extremely important (Smith et al., 2007). However, the information about this food source is not available at large scale.

The aim of this work is to fill this gap with a GIS-database approach that links together spatial and non-spatial information in order to map and measure fruit availability in the different periods of the year in the SE Alps, an important node of European migratory streams. We propose a new method to link non-spatial data about plant phenology with spatial open maps of vegetation as a starting point to deepen the knowledge of the bonds between migration and environment. The literature examination shows that the topic has been addressed by several authors (Berthold, 2001; Bauer, Ens & Klaassen, 2010; Bairlein, 2008; Chevallier et al., 2010) but very few studies have explored an holistic approach like the one we propose in this work.

The main goal of this study is to assess the availability of edible fruits in the late summer and autumn obtained creating coverage and richness maps for the plants that produce berries.

## Materials and Methods

### Study area

**Figure 1:**
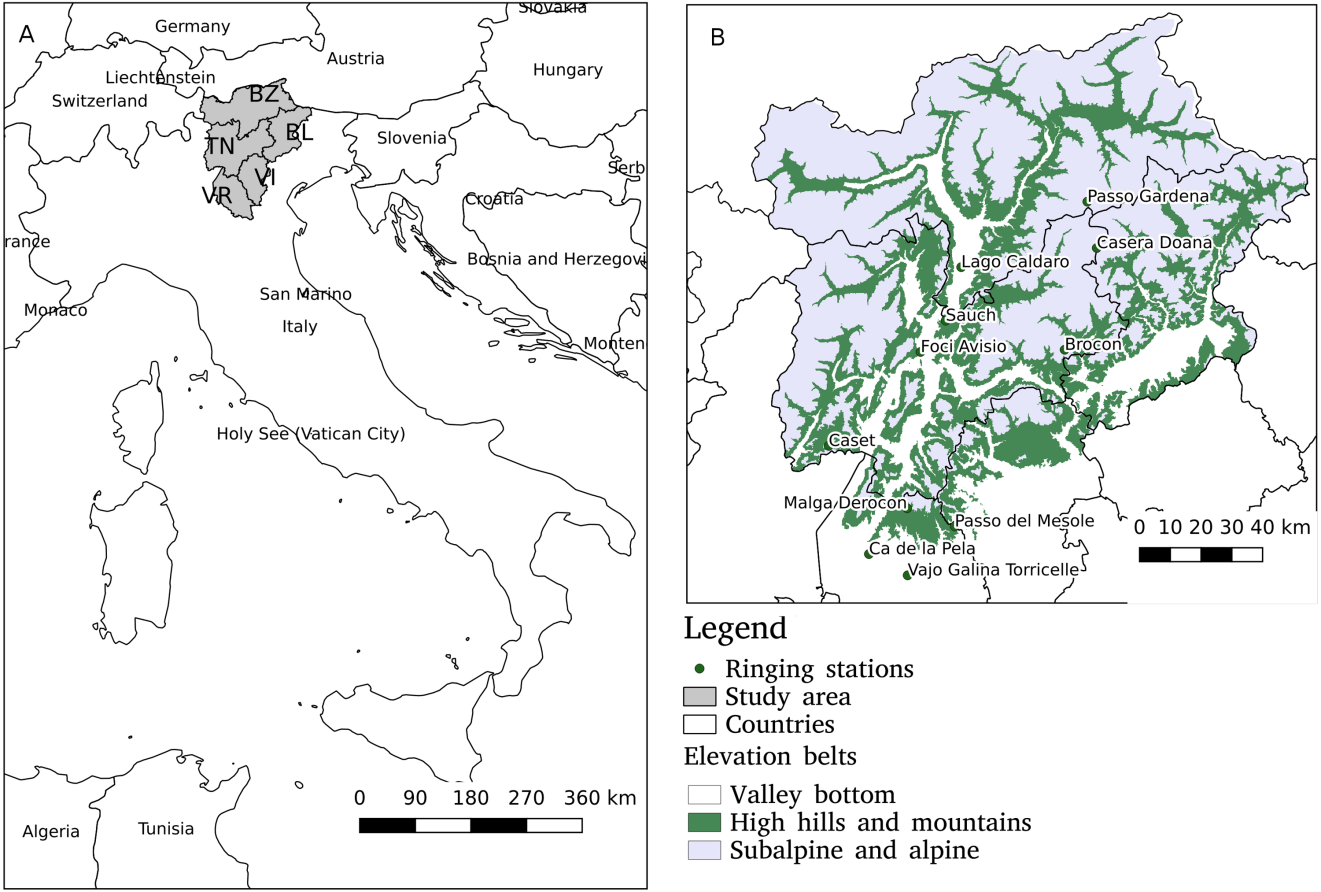
The study area encompasses five Provinces in NE Italy: BL, Belluno; BZ, Bolzano; TN, Trento; VR, Verona; and VI, Vicenza (A). The locations of the ringing stations and the three elevation belts are reported in the map B.

The study area (10.5°E, 45.2°N–12°E 46.5°) is located in the south-eastern Alps in Italy and includes the Provinces of Belluno, Vicenza and Verona (Veneto region) and the Provinces of Bolzano and Trento (Trentino Alto Adige region) see Fig. 1 and it covers about 21,000 km^2^. Belluno, Bolzano and Trento are mountainous area influenced by a continental climate, with most of the territory lying over 1,000 m above sea level, and around 55% covered by coniferous and deciduous forests (Ferretti et al., 2018). Vicenza and Verona Provinces are influenced by the Adriatic sea and have a larger extension of plain and hilly territories. Forests are present in 34% of Vicenza and 19% of Verona areas and intensive agriculture is present in the flat areas of both provinces.

The elevation range has been categorized according to three different altitude belts, the same used in Progetto Alpi (Pedrini et al., 2008) that are representative of different ecological environment:

Belt 1: Valley bottom and hilly area, at an altitude below 700 m, it occupies an area of 5,486.3 km^2^;

Belt 2: High hills and mountains, at an altitude between 700 and 1,400 m it occupies an area of 5,990.0 km^2^;

Belt 3: Sub-alpine and alpine, at an altitude of more than 1,400 m, is the larger area that occupies 10,177.7 km^2^. The spatial distribution of the three belts in the study area is shown in Fig. 1.

The five provinces represent a contiguous ecological system that connects Mediterranean areas with pre-alpine and alpine mountain environments. The area includes developed touristic, agricultural, industrial, and commercial areas that are connected through main road and railway transport infrastructures. The Provinces population in 2017 is around 208,000 in Belluno, 865,082 in Vicenza, 921.557 in Verona, 525,000 in Bolzano, 537,000 in Trento (http://www.istat.it). Most of the population is concentrated in the plain areas or in the valley floors. All these territories share a particular attention to mountain environment and forestry and they have translated this attention into scientific and practical management tools like detailed forest types catalogues Odasso (2002), Del Favero (2000), AA.VV (2010).

Diverse migration routes for many bird species have been identified in the study area. Since 1997 several ringing stations have been recording the bird passage in the frame of “Progetto Alpi” carried out by “ISPRA Istituto Superiore per la Protezione e la Ricerca Ambientale and MUSE Museo delle Scienze di Trento” (Pedrini et al., 2008). In the study area there are 11 ringing stations, located at different elevations (Fig. 1).

### Data collection

#### Plant phenology database

A list of 52 plant species that are commonly eaten by migratory birds (Snow & Snow, 1988; Jordano, 1982; Jordano, 1985; Hernández, 2009; Müller-Schneider, 1983) was produced (Table 1) and compared with the the standard dominance classification proposed by Blanquet reported in the forest type catalogue (Odasso, 2002). This document reports a detailed description of forest types composition. Braun-Blanquet scale is a recognized international standard for floristic and vegetation inventories Van Der Maarel (1975) and was converted in percentage of cover for the purpose of this study (see Table 2). We linked the ecological information about plant phenology with their occurrence in the Forest type maps and created a new geo-referenced database for the analysis.

**Table 1:** Summary of the fruiting time of the plants considered for the estimation of the fruit availability for migratory birds in the Alps.

Species	Months	Species	Months
* Vaccinium vitis-idaea*	7–9	* Cotoneaster nebrodensis*	9–10
* Vaccinium myrtillus*	7–9	* Rubus caesius*	8–10
* Sorbus aucuparia*	8–10	* Hedera helix*	9–11
* Rubus idaeus*	8–10	* Frangula alnus*	8–9
* Rosa pendulina*	9–10	* Cornus sanguinea*	8–9
* Sambucus racemosa*	7–10	* Rhamnus catharticus*	8–9
* Daphne mezereum*	9–10	* Prunus mahaleb*	7–8
* Berberis vulgaris*	9–12	* Rhamnus saxatilis*	8–9
* Juniperus nana*	9–10	* Empetrum hermaphroditum*	9–10
* Vaccinium gaultherioides*	9–10	* Viburnum opulus*	9–10
* Amelanchier ovalis*	8–9	* Rubus ulmifolius*	8–10
* Juniperus communis*	10	* Ribes petraeum*	9–10
* Lonicera nigra*	9–10	* Euonymus europaeus*	9–10
* Lonicera alpigena*	8–10	* Rosa arvensis*	8–9
* Viburnum lantana*	8–9	* Rosa villosa*	8–9
* Solanum dulcamara*	6–9	* Taxus baccata*	8–9
* Lonicera coerulea*	9–10	* Viscum album*	8–9
* Sorbus aria*	8–10	* Rubus ser.*	8–10
* Sambucus nigra*	8–9	* Cornus mas*	9
* Rhamnus pumilus*	8–10	* Cotoneaster integerrimus*	9–10
* Prunus avium*	6–7	* Rosa corymbifera*	8–9
* Prunus spinosa*	8–10	* Sorbus torminalis*	8–10
* Rosa canina*	9–10	* Prunus padus*	6–8
* Sorbus chamaemespilus*	9–10	* Rubus ser.*	8–10
* Crataegus oxyacantha*	8–10	* Rubus hirtus*	8–10
* Ligustrum vulgare*	8–9	* Rubus canescens*	8–10

**Table 2:** Braun–Blanquet (BB) original classification and description of plant abundance (columns 1 and 2) and percent of coverage (col. 3) used to estimate the fruit availability for migratory birds in the Alps.

BB class	Description	Coverage %
r	Single individual	0.5
+	Usually 2–5 individuals	1
1	More than 5 individuals and coverage less than 5%	5
2	Coverage range 5%–25%	25
3	Coverage range 26–50%	50
4	Coverage range 51–75%	75
5	Coverage range 76–100%	100

#### Plant occurrence data

Out of the list of 52 plants, the catalogue by Odasso (2002) provided the information about the coverage of 41 species. In order to obtain data for all the plants in the list, we accessed the data provided by the “Museo Civico di Rovereto” (MCR). This archive contains field records collected since 1989 using a systematic survey along transects and covers the entire study area (Dainese et al., 2017). This archive was designed to produce the local floristic atlas and latitude, longitude and elevation of each plant was obtained by GPS. The query of the database returned over 52,000 points of occurrence for all the 52 plant species identified.

**Figure 2:**
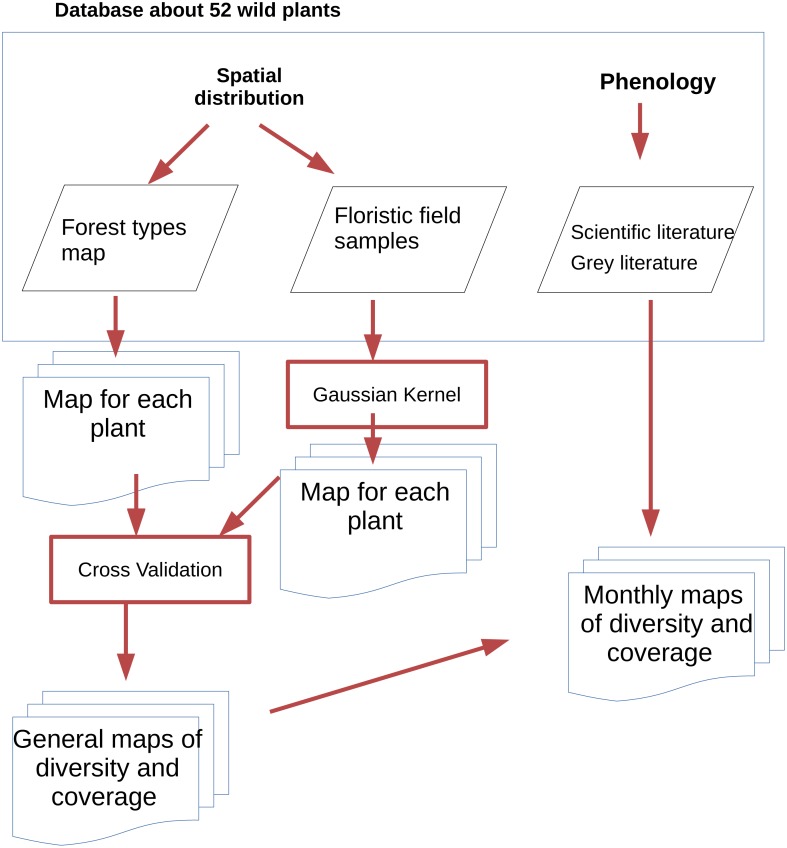
The multi disciplinary approach used in to produce the final maps combines various data sources to include spatio temporal aspects of wild berry fruitification.

#### Phenological maps of the plants

All of the 78 forest types available in the catalogue by Odasso (2002) were considered and the relative digital map was updated with the information coming from literature and interviews with experts to create a database integrating all the available ecological data. The forest types of Veneto (Del Favero & Lasen, 1993; Del Favero, 2000) and Alto Adige (AA.VV, 2016) followed a similar but not always matching nomenclature and some adjustment was required to match the types reported by Odasso (2002). The latter was chosen as a reference because of its detailed description of the vegetation composition in each forest type.

The flowchart of Fig. 2 describe the process used to create maps of continuous plant coverage and phenology. We used two different approaches to develop the maps according to the source: for the 41 of the species of plants carrying fruits that were reported in the forest type catalogue by Odasso (2002), we performed a spatial query on the above described database. The percent of coverage for 41, out of the 52 plants considered here, was available for each forest type from the study of Odasso (2002). Thus the cover value was processed joining the attributes in the table of the species with the forest types map and then converted into a single raster map for each plant. The coverage of the remaining 11 plants was modelled using a Gaussian Kernel (Xie & Yan, 2008) on the point data collected in the field. The sampling by MCR evenly covered the whole region and their number guaranteed the significance of statistic processing. This method was used to calculate the coverage of the 11 species that were not present in the forest type databases (Odasso, 2002): *Solanum dulcamara, Rhamnus catharticus, Rosa canina, Rhamnus pumilus, Empetrum hermaphroditum, Prunus spinosa, Ribes petraeum, Rosa arvensis, Rosa villosa, Viscum album, Prunus padus*. The kernel method was applied on the point derived from the systematic survey carried on by MCR to prepare the plant atlas of the area. For each of these 11 plant species a new raster map was generated processing Gaussian Kernel from point distribution. These maps were then aggregated following the forest types spatial distribution so they became comparable with the maps derived from forest types catalogue. Model validation and testing of the kernel algorithm were performed on the 41 plants that were available both in the forest types catalogue and in the point distribution format from MCR. The results were compared to the coverage reported in the Forest types catalogue (considered the ground truth) using Cohen Kappa parameter (Smeeton, 1985). Kappa parameter represents the accuracy rate of a classification. Both maps were reclassified following the same rules and then compared. The correspondence of kappa value is classified as following 0–20% insufficient, 21–40% scarce, 41–60% acceptable , 61–80% very good, 81–100% perfect. The output of this analysis is reported in the results.

The digital maps used in this work were: Vector maps of forest types (scale 1:10,000); vector map of geological structure (scale 1:10,000); Digital terrain model SRTM 90 m (Jarvis et al., 2008). All maps were downloaded from the sources cited at the end of the article if not stated otherwise (1. Provincia di Bolzano, http://geocatalogo.retecivica.bz.it/geokatalog/#!home; 2. Provincia di Trento, http://www.territorio.provincia.tn.it/portal/portale_geocartografico_trentino/254; 3. Regione Veneto, https://www.regione.veneto.it/web/agricoltura-e-foreste/banche-dati-cartografiche) and were georeferenced in datum 1940, international hellypsoid oriented in Monte Mario, projection Gauss-Boaga West fuse.

We used open source tools for the entire research process, from data analysis to editing (GRASS GIS (GRASS Development Team, 2008), QGIS (Quantum GIS Development Team, 2018), R (R Development Core Team, 2005), LaTeX, LibreOffice), following the philosophy highlighted by Rocchini & Neteler (2012) about Open source and Ecology. Environmental data where downloaded from open geo-portals (1. Provincia di Bolzano, http://geocatalogo.retecivica.bz.it/geokatalog/#!home; 2. Provincia di Trento, http://www.territorio.provincia.tn.it/portal/portale_geocartografico_trentino/254; 3. Regione Veneto, https://www.regione.veneto.it/web/agricoltura-e-foreste/banche-dati-cartografiche; 4. TM World borders, http://www.thematicmapping.org; Jarvis et al., 2008).

#### Migratory bird data

The flux of migratory birds was taken from the latest version of the report of “Progetto Alpi” that reported a standardised estimation of the ringed birds according to the actual number of days of activity of the station and size of the nets (Negra et al., 2003; Pedrini et al., 2008). The stations were open from mid-August to the end of October with a variable size of nets both across stations (range 105–1,300 square meters) and within the station over time. The captures were reported on a five day basis, or *pentade*, the fixed-date system proposed by Berthold (1973), considered the standard interval in bird migration research. Some stations collected data across the operative season at least once in each pentade, others every day in any odd pentade, others more irregularly (Negra et al., 2003). A grand total of 83 species of birds was reported in the four ringing stations of the study area for a grand total of 8915 captures (Negra et al., 2003).

We extracted from the above cited reports the list of birds captured at each station over a five day scale and classified the wildfowl according to the feeding guild devised by Waldenströrm et al. (2002). For the purpose of this study we later simplified it into three guilds: Granivores, Insectivores, Berry eaters and Raptors, the last one was not considered in this study. We retained the Granivores guild as it was in the article by Waldenströrm et al. (2002), while the Insectivores class were split in two: we labelled “Insectivores” the birds that are strictly insectivores and “Berry eaters” the other insectivore ones known to eat also fruit and berries based on literature (Snow & Snow, 1988; Jordano, 1982; Jordano, 1985; Hernández, 2009; Müller-Schneider, 1983; Sibley & Ahlquist, 1990). This list is available as Table S1, only Passerines were used for this study.

The number of birds ringed every five days step was standardized into a Capture Index (number of captures per day per 250 square meters of net), in order to account for the number of active days in the five day interval and the varying size of the nets:

\begin{eqnarray*}\text{CI}=\text{Capture Index}=N~of~captures/(days~of~activity~per~pentade\ast square~meters\nonumber\\\displaystyle \quad \quad of~net/250) \end{eqnarray*}

In this work we calculated the Capture Index for each feeding guild instead of using the cumulative CI available in Negra et al. (2003). In order to overlap the pendade system with the calendar, we converted the pentade system into decimal number, considering that each month had six “5 day intervals”, thus each pentade is 0.16 months. The proportion of capture and CI were later averaged per month for each guild in order to compare the fluxes of the different feeding types with the monthly maps of plant phenology.

## Results

The database of plants we created allowed us to assess the number of species carrying fruits for each forest type and to create a raster map (cell size 100 m) of coverage for each species of plant reported in Odasso (2002). The percent of coverage for each plant species for each forest type is reported in Table S2. In this way it was possible to highlight in which forest types the specific richness was higher. The forest types where richness accounted for more than 20 species were 12; between 10 and 20 species were 20; the remnant types had less than 10 species. In general, formations with beech and pine were the richest, while spruce and larch formations had less species as reported in Table 3.

**Table 3:** Forest types hosting more than twenty (first column) or more than 10 (second column) species of plants producing berries.

More than 20 species	10–20 species
Calciphilous silver fir forest with European beech	Silver fir forest of fertile soils
Acer and small-leaved lime forest	Acer and ash forest
European beech forest with *Dentaria*	Acer and ash forest with alder
European beech forest with European hop-hornbeam	European beech forest with conifers
European beech forest with European yew	Siliceous European beech forest with *Luzula sp.* or grasses
Alpine dwarf mountain pine scrub with *Erica sp.*	European larch forest (secondary succession)
South European flowering ash and European hop-hornbeam forest	European larch and arolla pine forest with rhododendron
Oak-European hop-hornbeam forest	Xeric European larch and arolla pine forest with juniper
Montane xeric Norway spruce forest	Evergreen oak forest with hop-hornbeam
Scots pine forest with beech	Evergreen oak forest with turpentine tree
Scots pine forest with Austrian pine	Alpine dwarf mountain pine scrub with rhododendron
Pine forest (pioneer formation)	Alpine dwarf mountain pine scrub invasive of former pastures
	Montane Norway spruce forest
	Norway spruce forest with *Erica spp.*
	Sub-alpine Norway spruce forest
	Norway spruce forest (secondary succession)
	Scots pine forest with Norway spruce
	Scots pine forest with South European flowering ash
	Xeric Scots pine forest
	Sessile oak (or Turkey oak) forest

The list of forest types was simplified in order to preserve the document readability: since the Italian name of the forest types uses the common names we translated them using the common names as well. Table S3 reports the Italian name of the forest types and further information such as Natura 2000, Syntaxon name and Eunis codes corresponding to each type when available. For detailed description of the types please refer to Barbati et al. (1999), Lasen (2006) and AA.VV (2016), and the literature cited in the paper.

Gaussian Kernel was calculated for all the plants in order to estimate the accuracy of the Kernel method on the species from the forest type catalog that was considered the ground truth. The Kappa parameter for these 41 species was always above 80% except for a few very common herbs that are broadly spread also in open areas such as *Vaccinium myrtillus*, in that case the value was around 50%. Since the 11 plant species for which the coverage was not reported by Odasso (2002), are quite rare or have a limited distribution, the method was considered applicable and the results satisfactory. With this approach we were able to estimate the coverage of the remaining 11 species in each forest type.

**Figure 3:**
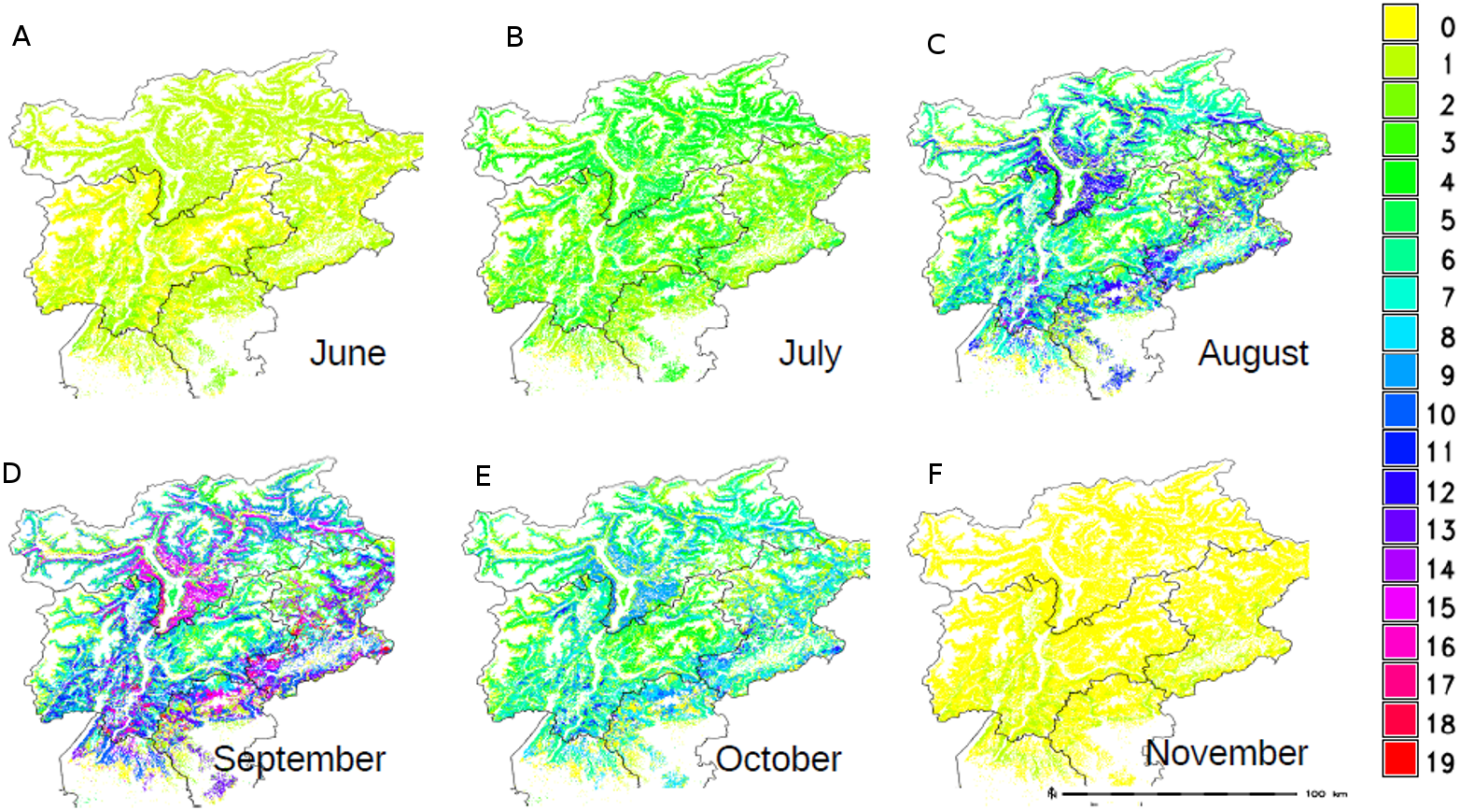
Monthly specific richness map: number of plants species carrying ripen fruits that are edible by birds, in each forest type of NE Italy.

**Figure 4:**
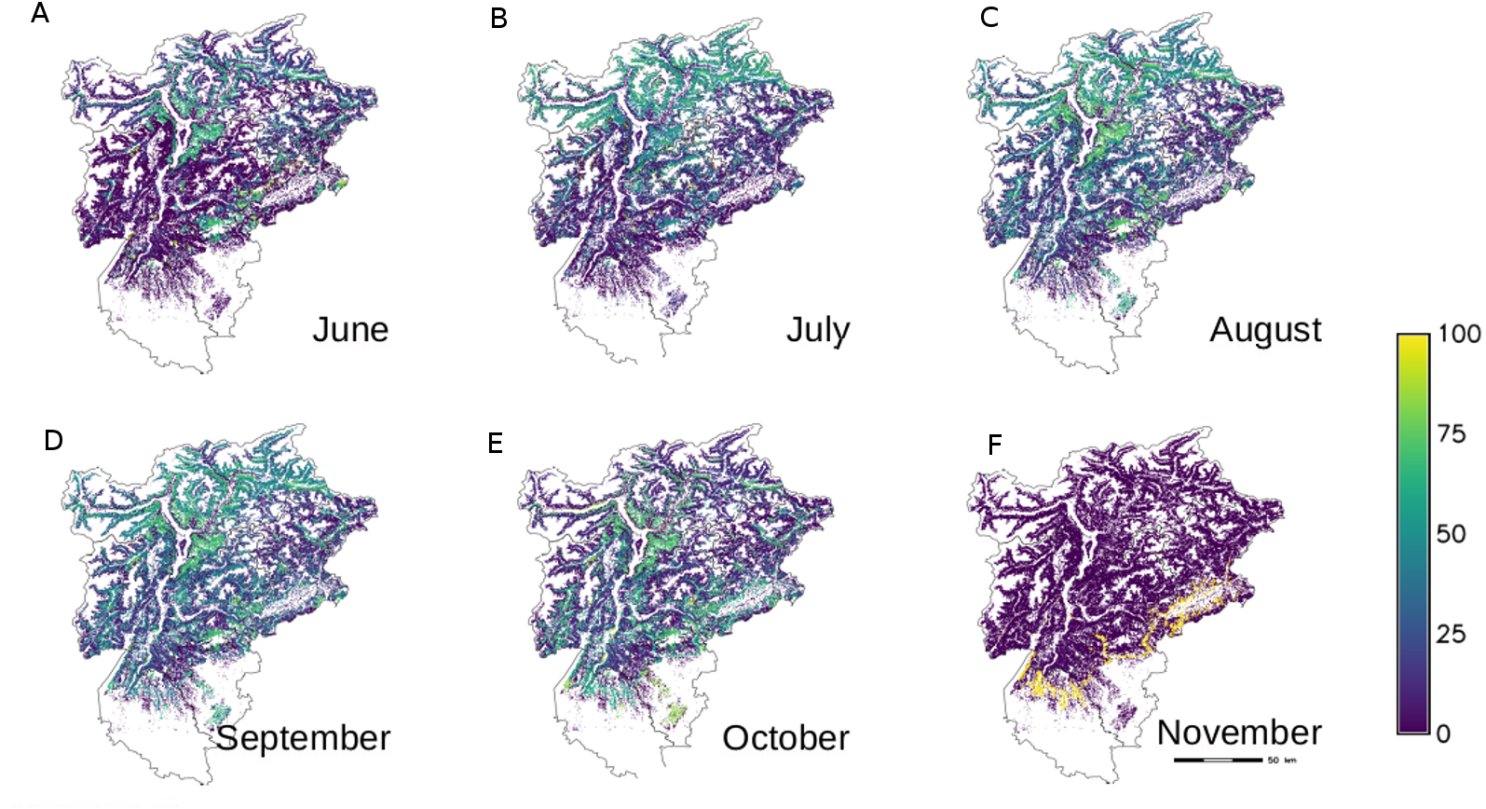
Monthly coverage of plants carrying ripen fruits edible by birds in each forest type of NE Italy.

The maps of the single plant species, either obtained from spatial queries or from modelling, were combined to obtain the following results: total specific richness map (not shown), the total number of species occurring in each forest type; monthly specific richness map (Fig. 3) i.e., the number of species aggregated according to the fruitification period in the different months as reported in Table 1; monthly coverage with the sum of the each species coverages aggregated depending on the fruitification period in the different months (Fig. 4).

A great extent of the study area is covered by an average number of plant species, while few areas show a very high or very low richness level. The bigger surface is interested by a specific richness of 7, 10 and 15 species while the average is about 9 species per square kilometre.

The monthly cover maps have been reclassified using the Braun-Blanquet coefficients reported in Table 2. This classification is generally used for each species and not for their aggregations, but we used this classification to maintain coherence with the single species map. The reporting tools of GRASS GIS were used to quantify the values of richness and coverage per month: results are shown in Table 4. The months of August, September and October showed the highest values, with some forest types entirely covered in berries, in perfect timing with the migratory fluxes observed in the study area (Pedrini et al., 2008). From August to October more than half of the investigated area offers mature fruits and berries granting a diffused food availability (Table 4).

**Table 4:** Summary of the monthly values for richness and coverage of fruiting berries in NE Alps: N. Max and Max coverage are referred to a single forest type. For instance, in June there are only two species carrying fruits and they cover at maximum 50% of a forest type unit. Overall the area interested by these two plants is 7,522 km^2^ i.e., 34% of the study area.

Month	N. Max	Max coverage per forest type %	Area km^2^	% of study area
June	2	50	7,522.53	34.74
July	7	50	9,621.40	44.43
August	16	100	9,935.26	45.88
September	20	100	9,990.26	46.14
October	12	75	9,436.64	43.58
November	1	0	556.51	2.57

**Figure 5:**
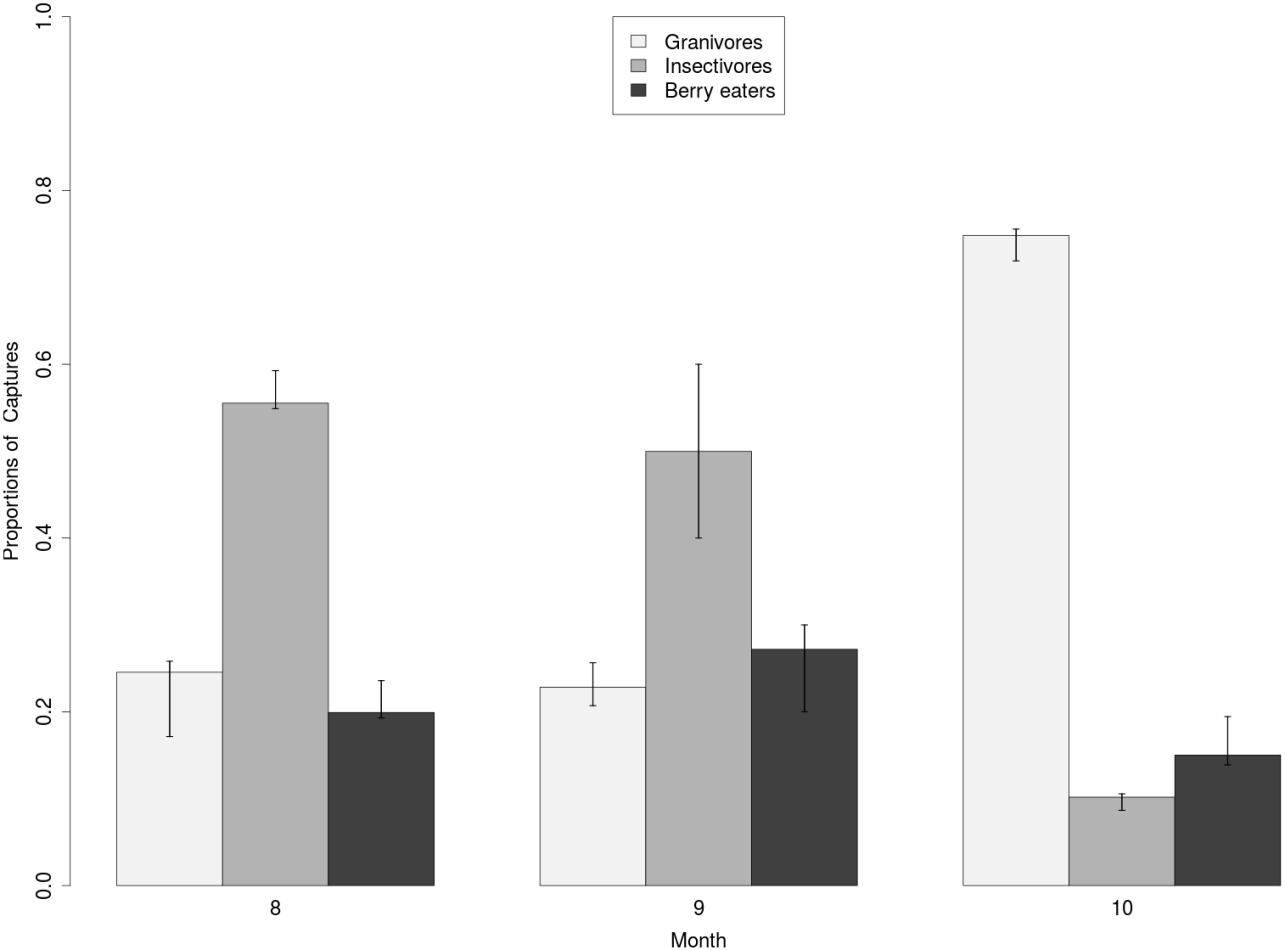
Proportion of the captures of migrant birds per month in NE Italy according to different feeding guilds. Bars represent the average value obtained from the data reported by Negra et al. (2003) and Pedrini et al. (2008), segments represent the standard error interval. In September, the peak in the capture of berry eating birds coincides with the maximum availability of fruits.

The average capture rate for migrant birds show a different pattern for the three feeding guilds considered: the plot of Fig. 5 summarises the flux of migrant birds in the study area recorded by “Progetto Alpi” (Negra et al., 2003; Pedrini et al., 2008), standardized per effort in terms of days and net surface and then aggregated on a monthly basis. Despite Berry eaters are not the most represented in terms of individuals, accounting for 20%, 30% and 15% of the monthly captures in August, September and October respectively, data show that their passage reaches the highest point in September. On the other hand, granivore birds are the most abundant in October, reaching up to 70% of the individuals, in correspondence to the time of seed production by trees and . Finally, the majority of strictly insectivores pass in August and September with nearly 55% of the captured individuals (Fig. 5), but in October their presence in the nets drops to 12%.

### Elevation range of fruit availability

**Figure 6:**
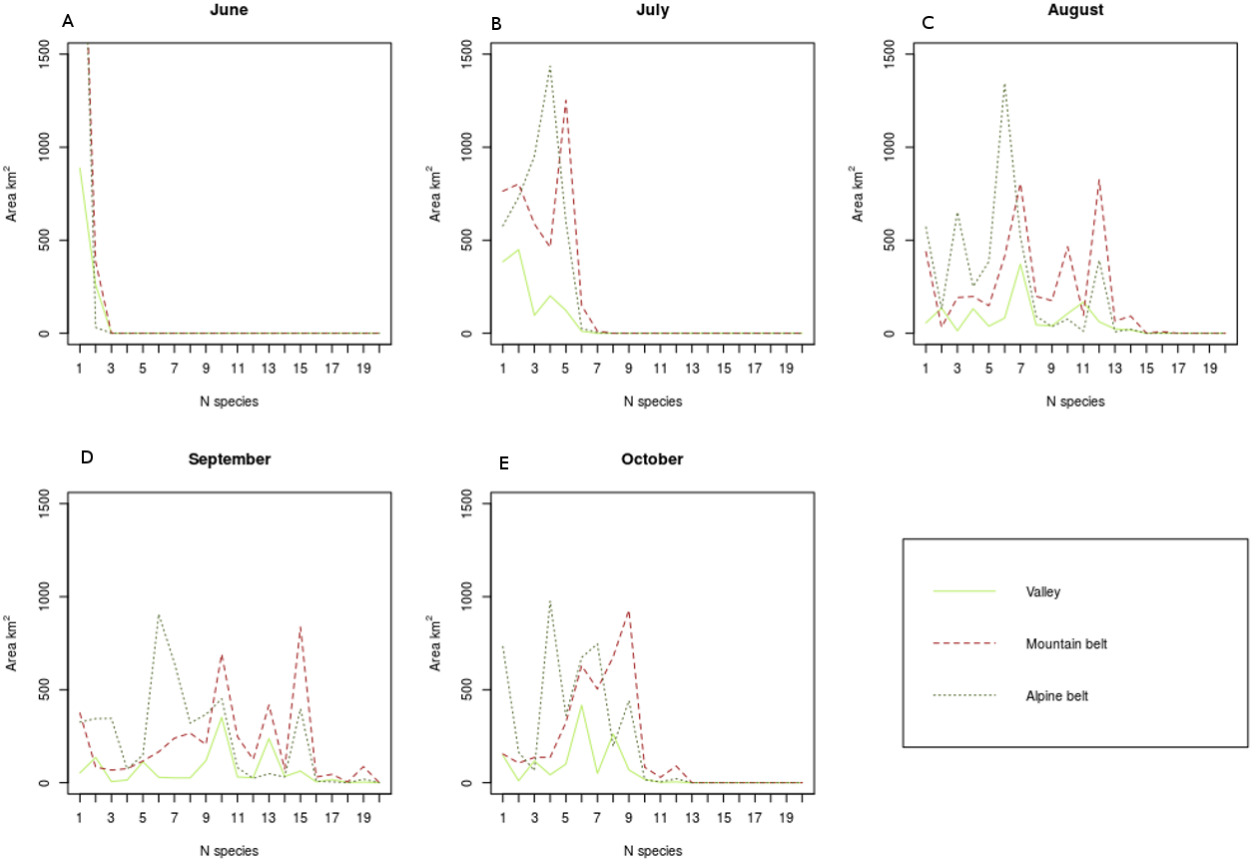
Area covered by plants carrying fruiting berries over time according to the elevation belts in the SE Alps.

Fruiting species richness and cover have been evaluated also in relation to three different altitude belts, the same used in Progetto Alpi (Pedrini et al., 2008). The number and coverage of fruiting plants increase from June to September, where there is a peak in the values, then decrease in October and reach a minimum in November, as illustrated in Fig. 6. This phenomenon occurs generally across the study area, but in the high hills and mountain belt the species richness and surface covered by fruiting plants are always higher than at lower and upper elevations.

**Figure 7:**
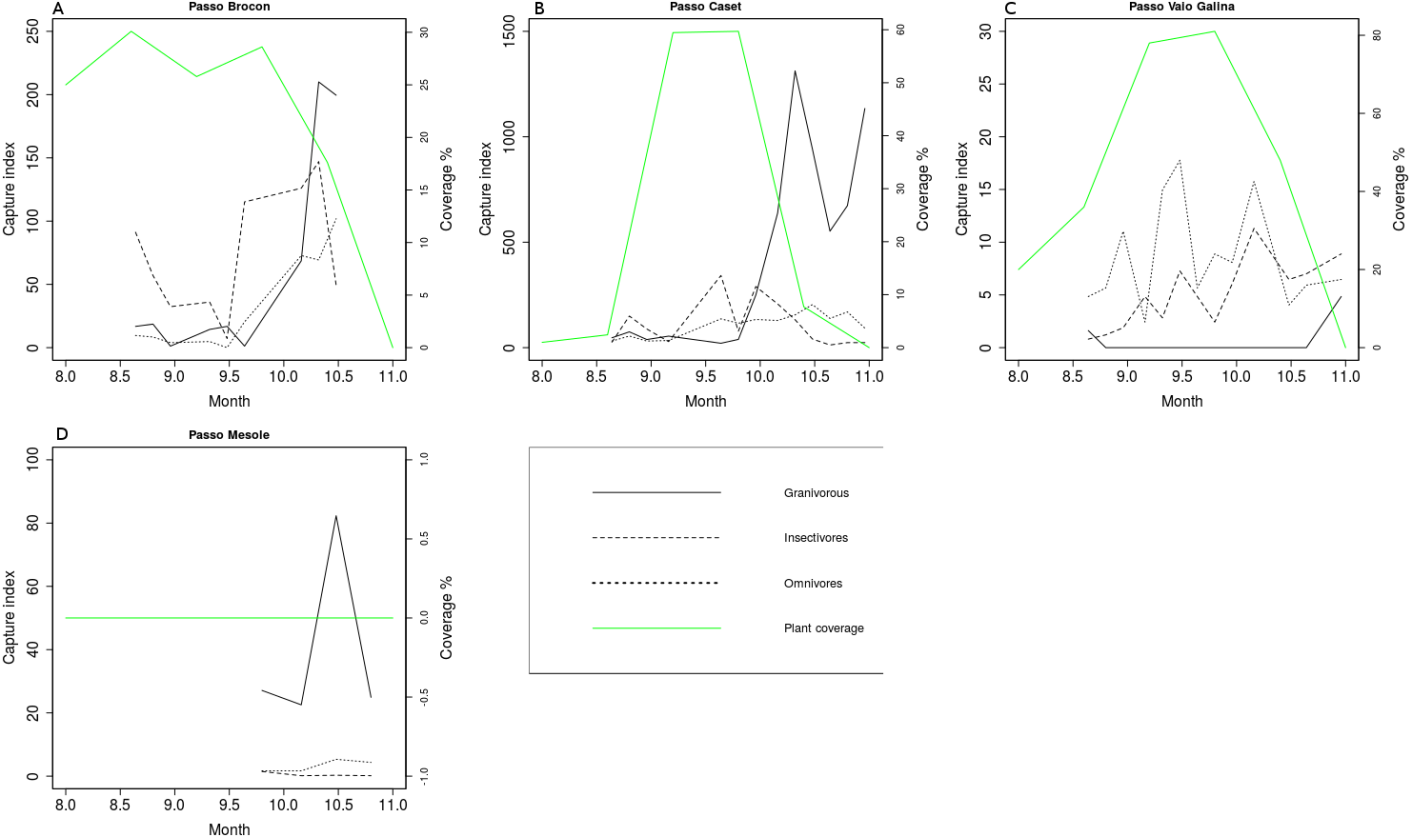
Capture index (Number of ringed birds standardized by effort and size of the nets) according to simplified feeding guild and berry coverage at selected ringing stations in Italian Alps.

The species of birds captured at each station were classified into simplified feeding guilds: Granivores (18 species, 5,307 ringed birds), Insectivores (32 species, 1,853 ringed birds), Omnivores (24 species, 1,727 birds). About 80% of the captured species of birds were Omnivores, in the sense of berry eater, either during migration or throughout the year while the most captured feeding guild in term of individual was the granivores one. For each station we standardized the number of captures of each simplified feeding guild into a Capture Index and we overlapped the trends with the fruit coverage of the relative ringing station (Fig. 7). The graphs in Fig. 7 show that the peak of fruit availability from mid September to mid-October co-occurs with the peak of captured insectivores and omnivores species, while the peak of the passage of granivore birds occurred in mid-late October.

## Discussion

The organization and harmonisation of the information present in various sources allowed the production of 16 maps, accounting for all the 52 species considered, that spatialise for the first time the available knowledge about presence, coverage and fruiting phenology. The database structure is flexible and could be expanded to include other kind of data and analysis and the open tools used allows the repeatability of the method for other species and context. The procedures that MCR uses to collect floristic data are the standard sampling procedures to create plant atlas that are a standard used in all Europe (Dainese et al., 2017). The tables provided in the supplemental material can be used to reclassify forest types of different areas of Europe: in fact, Table S3 reports the conversion between the forest types and the codes of Natura2000, Eunis habitat code (level 4/5) and Corine Palearctic equivalent. Therefore, the method we developed should be easily replicated in other contexts. The bottleneck is finding data from multiple sources and grey literature such as local technical reports and experts opinion.

Results about the coverage for the single species of plants showed a good correspondence between the percentages of coverage expected and those obtained from the processing of the floristic point sampling which has allowed us to harmonize data of different nature. The cartographic production of this work includes six monthly maps of the cover reclassified according to Braun-Blaquet and six monthly maps of species richness. In addition a single map of the plant richness summarize in a single geo-referenced source all the material collected for this study. The maps confirms the lower availability of wild berries in the man-made environment of the valleys. On the other hand, the intermediate elevation belt has the greatest fruit availability both in terms of the extent and of species richness and is probably the most exploited by migratory birds that move along the direction of the main valleys.

Concerning the spatial distribution of the edible berries, we were able to identify and map the beech and pine formations as the richest and to analyse the distribution along the elevation ranges and time. The valley floor is occupied by a few fruiting species present on a rather limited surface (Fig. 6). In fact, this belt is both the smallest and the most exploited with intensive agriculture, urban areas and infrastructures. Localized zones with a higher berry richness are located on the hillside at the border with the next elevation belt, but in general most of this belt is occupied by anthropic environments. The bottom valley of the study area is intensively cultivated with apple and vineyards, where the new intensive trellising systems affects negatively the presence of birds (Assandri et al., 2017). Despite the fact that grapes can be a good source of food for birds, the current management and the limited time during which the fruits are available before the harvest, reduce the accessibility as food source for migratory birds.

The high hills and mountains horizon is the belt of greatest richness of fruit bearer plants: in fact, in some months, they cover up to 80% of the area. This belt is almost entirely occupied by forests and there are some areas where locally the variety of species is high (15–20 species of interest in the same forest type) and very high (over 20 species). The sub-alpine and alpine belt has more than half of the territory above the tree line, where no information was found regarding edible plants, therefore these analyses refer only to the forested areas. The area of the territory containing the plants of interest as well as the trend of the curve are similar to that of the intermediate band, but with the curve maximum in correspondence with a lower number of species (Fig. 6). As summer advances, the availability of plants in fruit in all the belts increases reaching a peak in September. In September, in all the three altitude belts, forest types are covered for more than 50% by fruiting plants. As autumn advances, in October the trophic availability decreases in all belts, with a steeper drop in the Subalpine and Alpine range.

In the study area the peak of production of berries happens in September and co-occurs with the peak of migration of insectivore and berry eating birds, whereas the availability of berries decreases in October in coincidence with the peak of granivore birds. However, October is the month in which some species of tree such as beech and common alder produce seeds, that are eaten by birds such as chaffinch, brambling and hawfinch. This study focused on the production of berries, but the maps of tree seeds can add more insights about the food availability for migrant birds. The inclusion of seed production by all the trees in the study area could explain the peak of captures of granivore birds in late October, that represent the majority of passing individuals. Despite this limit, about 80% of the captured species of birds are berry eaters (Negra et al., 2003), and our results focused on this feeding guild.

## Conclusions

This work presents a multy-disciplinary study about bird migration encompassing spatialisation of fruit availability, reports from ringing stations and experts opinion of vegetation scientists. The availability of detailed forestry data and the possibility to retrieve those map from public agencies proved critical to develop this work. The creation of a unique database including the presence and periods of fruitification of 52 species of plants and a series of original cartographic themes provided a synthesis of the knowledge available in the area through mixed techniques and interpretation of ecological data.

The conservation and management of stopover sites that can provide energy resources for migratory step is of particular importance for the protection of migration (Smith et al., 2007; Sapir et al., 2004). The results of this work provide very detailed information about location and phenology of the plants that produce berries and fruits, in addition, the maps can be be used to identify suitable locations to displace nets and to better understand the stopover behaviour across the study area (Cantiani et al., 2016). All these considerations suggest a management of stopover areas which also includes vast bushy areas able to provide a suitable habitat for insects. The protection of plant diversity definitely has a positive role in supporting also the invertebrates community, but surely this is a theme to be explored in further studies.

Bird migration is a complex natural phenomenon, that is affected by a great number of environmental, climatic and species specific variables for flying animals. The results obtained on the assessment of the temporal and spatial berries availability are useful not only for birds (resident or migratory) but also for other species, as well as a starting point for further studies and to support decision-making processes in environmental planning. These maps can also be used for assessing fruit availability for other frugivores.

##  Supplemental Information

10.7717/peerj.6394/supp-1 Table S1 List of bird species and their feeding guild

10.7717/peerj.6394/supp-2 Table S2 Percent of coverage of each species per forest types (see also Table S3)

10.7717/peerj.6394/supp-3 Table S3 Correspondence between forest types and other official coding systems

10.7717/peerj.6394/supp-4 Supplemental Information 1 Raster maps of wild berry plants and their metadata Please refer to the README.txt file for detailed descriptions of maps and metadata.

## Additional Information and Declarations

### Data Availability

The following information was supplied regarding data availability: The raw data are available in the Supplemental Files.
