# Supplementary material for: Fruit availability for migratory birds: a GIS approach
Source: PeerJ. 2019 Feb 5;7:e6394. doi: 10.7717/peerj.6394 (PMC6368004; doi:10.7717/peerj.6394)
Supplement: Table S3 [file peerj-07-6394-s003.pdf]

Supplemental Table 3 Tattoni et al. 2018 Fruit availability for migratory birds: a GIS approach PeerJ

Correspondence of the main forest types used in this paper (Odasso 2002) with the Reference phytosociological associations (to be considered as a guidance), Habitat code of Natura 2000 and Eunis habitat code (na means code non available) according to (Barbati et al 1999, AA.VVV 2010). The last column reports the subtype codes used in this work to assess plants richness and coverage for each cover subtype which differs from the main type depending on to geological substrate and forest stand parameters. These latter codes match with the ones reported in Supplemental Table1.

Forest Type name in Italian	(Reference phytosociological associations) Syntaxonomy	Natura 2000 habitat code	Eunis habitats (level 4/5) and Corine Palearctic equivalent	Subtypes included
Orno - ostrieto tipico	Seslerio albicantis-Ostryetum carpinifoliae Lausi et al. 1982 corr. Poldini et Vidali 1995 (= Seslerio variae-Ostryetum carpinifoliae Lausi et al. 1982, Art.43)	na	G1.H/P-41.81	OO_mi, OO_ca, OO_si
Orno - ostrieto primitivo	Hemerocallido lilioasphodelo-Ostryetum carpinifoliae Poldini 1982 and Cytisantho radiatae-Ostryetum carpinifoliae Wraber 1960 and Seslerio albicantis-Ostryetum carpinifoliae detritica Lausi et al. 1982 corr. Poldini et Vidali 1995 (= Seslerio variae-Ostryetum carpinifoliae Lausi et al. 1982, Art.43)	na	G1.H/P-41.81	OO_PRI
Castagneto – robinieto	p.p. Carici umbrosae-Quercetum petraeae (Poldini 1982) ex Marinek 1994, Quercetosum petraeae subass. (Poldini 1982) ex Marinek 1994; p.p. Buglossoido purpurocaeruleae-Ostryetum carpinifoliae Gerdol, Lausi, Piccoli et Poldini 1982	9260	G1.B/P-41.9	CS_RO
Aceri – frassineto	Hacquetio epipactido-Fraxinetum excelsioris Marinek 1990 ex Poldini et Nardini 1993 var. geogr. Anemone trifolia Poldini et Nardini 1993 (= Carpino betuli-Fraxinetum excelsioris Poldini 1982 non Duvigneaud	9180	G1.A/P-41.39	AF
Aceri – frassineto con ontano	p.p. Hacquetio epipactido-Fraxinetum excelsioris Marinek 1990 ex Poldini et Nardini 1993 var. geogr. Anemone trifolia Poldini et Nardini 1993 (= Carpino betuli-Fraxinetum excelsioris Poldini 1982 non Duvigneaud 1969 cerastietosum sylvaticae Poldini 1982)	9180	G1.A/P-41.39	AF_OB
Aceri – tiglieto	Ornithogalo pyrenaici-Carpinetum betuli Marinek, Poldini et Zupani 1983, Tilietosum platyphylli subass. prov. in sched.	9180	G1.A/P-41.39	AT_ca
Aceri – tiglieto	Ornithogalo pyrenaici-Carpinetum betuli Marincek, Poldini et Zupancic 1983, fraxinetosum excelsioris subass. Poldini in sched. var. Tilia	9180	G1.A/P-41.39	AT_mi, AT_si
Pineta xerica endalpica	Erico carneae-Pinetum sylvestris Br.-Bl. in Br.-Bl. et al. 1939 nom. Inv.	na	G3.3/P-42.32	PS_XER_ca, PS_XER_ca, PS_XER_mi, PS_XER_si,
Pineta pioniera	"Erico carneae-Pinetum sylvestris Br.-Bl. in Br.-Bl. et al. 1939 nom. Inv. vArctostaphylos uva-ursi"ar.	na	G4.4	PS_PRI
Pineta tipica con abete rosso	p.p. Anemono trifoliae-Fagetum sylvaticae with spruce Tregubov 1962 var. geog. Luzula nivea Marincek et al. 1989	na	G3.2/P-42.22	PS_AR
Pineta con faggio o specie nobili	p.p. Anemono trifoliae-Fagetum sylvaticae Tregubov 1962 var. geog. Anemone trifolia Marincek et al. 1989	na	G3.6/P-42.61	PS_FA
Pineta igrofila	Fraxino orni-Pinetum nigrae Martin-Bosse 1967 pinetosum sylvestris subass. Lasen et Poldini 1989	na	G3.6/P-42.61	PS_IGR
Pineta con orniello	Fraxino orni-Pinetum nigrae Martin-Bosse 1967 pinetosum sylvestris subass. Lasen et Poldini 1989	na	G3.6/P-42.61	PS_OR_ca, PS_OR_mi, PS_OR_si, PS_PN,
Pecceta secondaria o sostitutiva	Carex alba-Picea phytocoenon	na	G3.3/P-42.34	PEX
Faggeta con carpino nero	p.p. Hacquetio epipactido-Fagetum sylvaticae Kosir 1962, var. geogr. Anemone trifolia Kosir 1979, Luzula nivea subvar. Geogr. Poldini et Nardini 1993	9150	G1.7/P-41.13	FA_CN
Faggeta silicicola a luzula o graminacee	Anemono trifoliae-Fagetum sylvaticae Tregubov 1962 var. geog. Luzula nivea Marincek et al. 1989, caricetosum albae subass. Marincek et al. 1989	9110	G1.7/P-41.16	FA_GRA
Faggeta mesalpica con conifere	Anemono trifoliae-Fagetum Tregubov 1962 var. geog. Luzula nivea Marincek et al. 1989	9130	G1.7/P-41.13	FA_CON
Faggeta altimontana	Dentario pentaphylli-Fagetum sylvaticae H. Mayer et Hofmann 1969, fagetosum subass.Poldini et Nardini 1993, altitudinal life form.	9140	G1.7/P-41.15	FA_ALT
Faggeta tipica a dentarie	"p.p. Dentario pentaphylli-Fagetum sylvaticae H. Mayer et Hofmann 1969"	9130	G1.7/P-41.13	FA_mi, FA_TA, Fa_si

Forest Type name in Italian	(Reference phytosociological associations) Syntaxonomy	Natura 2000 habitat code	Eunis habitats (level 4/5) and Corine Palearctic equivalent	Subtypes included
Abieteto calcicolo con faggio	Cardamino pentaphylli-Abietetum albae H. Mayer 1974 nom. mut. Em. Gafta 1994	9130	G3.1/P-42.13	AB_FA
Abieteto dei suoli fertili	Adenostylo glabrae-Abietetum albae H. Mayer et Hofmann 1969 em. Gafta 1994	9130,9410	G3.1/P-42.12	AB_ca
Abieteto dei suoli fertili	Cardamino pentaphylli-Abietetum albae H. Mayer 1974 nom. mut. Em. Gafta 1994	9130,9410	G3.1/P-42.13	AB_mi, AB_si
Abieteto silicicolo dei suoli acidi	Luzulo nemorosae-Piceetum abietetis (Schmid et Gaisberg 1936) Br.-Bl. et Siss. in Br.-Bl. et al. 1939 (=Luzulo-Abietetum Oberd. 1957)	9130,9410	G3.1/P-42.13	AB_GRA
Mugheta a rododendro ferrugineo	Rhodothamno-Rhododendretum hirsuti (Aich. 1933) Br.-Bl. et Siss. in Br.-Bl. et al. 1939	4060	F1.2/P-31.51	MU_FR
Mugheta a erica	Erico carneae-Pinetum prostratae Zöttl 1951 nom. Inv.	4070	F1.2/P-31.51	MU_ER, MU_SC
Lecceta con carpino nero	Fragments of Quercion ilicis Br.-Bl (1931) 1936	9340	G2.1/P-45.31	LE_CN, LE_TB
Mugheta a rododendri	p.p. Rhododendro ferruginei-Pinetum prostratae Zöttl 1951 nom.inv.	4070	F1.2/P-31.52	MU_ROD
Ontaneta di ontano verde	Alnetum viridis Br.-Bl. 1918	na	na	OA
Pecceta a erica con pino silvestre	Erico-Piceetum or Carex alba-Picea phytocoenon	9410	G3.2/P-42.22	PE_PS_ca, PE_PS_ca, PE_PS_mi, PE_PS_si ,
Pecceta a megaforbie con ontano verde	Adenostylo alliariae-Abietetum albae Kuoch 1954 (= Adenostylo alliariae-Piceetum Hartm. 1942)	9410	G3.2/P-42.21	PE_OA
Pecceta altimontana tipica	Luzulo nemorosae-Piceetum abietetis suoli mesici altimontana (Schmid et Gaisberg 1936) Br.-Bl. et Siss. in Br.-Bl. et al. 1939 (= p.p. Homogyno-Piceetum, Luzuletosum albidae subass. Zukrigl 1973)	9410	G3.2/P-42.21	PE_mi
Pecceta subalpina	Luzulo nemorosae-Piceetum abietetis (Schmid et Gaisberg 1936) Br.-Bl. et Siss. in Br.-Bl. et al. 1939 (= p.p. Homogyno-Piceetum, Luzuletosum albidae subass. Zukrigl 1973).	9410	G3.2/P-42.21	PE_SUB_mi, PE_XER_si, PE_SUB_mi, PE_SUB_si,
Ostrio – querceto	Buglossoido purpurocaeruleae-Ostryetum carpinifoliae Gerdol, Lausi, Piccoli et Poldini 1982	na	G1.H/P-41.81	OQ
Pecceta subalpina	Larici-Piceetum abietetis (Br.-Bl. et al. 1954) Ellenberg et Klötzli 1972 (= Homogyno	9410	G3.2/P-42.21	PE_SUB_ca
Pecceta igrofila a sfagni o molinia	Sphagno girgensohonii-Piceetum abietetis Kuoch 1954	9410		PE_SF
Pecceta altimontana tipica	Adenostylo glabrae-Piceetum abietetis M. Wraber 1966 ex Zukrigl 1973	9410	G3.2/P-42.21	PE_ca
Lariceto tipico a rododendro	Asplenio viridis-Laricetum H. Mayer 1984	9420	G3.3/P-42.34	LA_RF, LA_XER_ca, LA_XER_mi, LA_XER_si, LAX
Lariceto con ontano verde	Calamagrostio villosae-Pinetum cembrae Filipello, Sartori et Vittadini 1981, alnetosum viridis subass. Filipello, Sartori et Vitadini 1981	9420	G3.3/P-42.32	LA_OA
Larici – cembreta tipica a rododendro	Calamagrostio villosae-Pinetum cembrae Filipello, Sartori et Vittadini 1981, Seslerietosum albicans subass., Filipello, Sartori et Vitadini 1981	9420	G3.3/P-42.32	LC_RF, LC_XER_ca, LC_XER_mi, LC_XER_si,
Lariceto secondario o sostitutivo	Rhodothamno chamaecisti-Laricetum H. 1984 Mayer	na	G4.4	LAX
Ontaneta di ontano nero	p.p. Aceri-Fraxinetum s.l.	91E0	G1.1/P-44.21	, OB, ON
Querceto di rovere (o cerro)	Carici umbrosae-Quercetum petraeae (Poldini 1982) ex Marincek 1994	91L0,9170	G1.8/P-41.26	QR, QC_CA
Formazioni transitorie	Transition shrubland	na	na	TR
