# Supplementary material for: Fruit availability for migratory birds: a GIS approach
Source: PeerJ. 2019 Feb 5;7:e6394. doi: 10.7717/peerj.6394 (PMC6368004; doi:10.7717/peerj.6394)
Supplement: Supplemental Information 1 — Please refer to the README.txt file for detailed descriptions of maps and metadata. [file peerj-07-6394-s004.zip › Supplemental_Table_1.pdf]

Supplemental Table 1 Tattoni et al. 201X Food availability and flight routes for migratory birds: a GIS approach PeerJ N.XXXXXX

List of birds captured in the 4 ringing stations considered in the manuscript taken from the official report by Pedrini and Spina (2002). For each species is reported the feeding guild according to (Waldenstroem et al 2002) Guilds: A, raptors; B, ground-foraging invertebrate feeders; C, ground-foraging granivores; D, ground-foraging insectivores; E, arboreal insectivores; F, aerial insectivores; G, reed- and herbaceous plant-foraging insectivores; H, shoreline-foraging invertebrate feeders; I, aquatic invertebrate feeders; J, plant-eating species; K, fish-eating species; L, opportunistic feeders.

A simplified guild also reported, for the purpose of this paper , that takes into account the feeding behavior during the migration , so some insectivores that rely on berries were labeled as omnivores (Jordano 1982, 1985, Müller 1983, Brandle 2002 , Snow and Snow, 1988,, Hernandez, 2009, Muller-Schneider, 1983)

**Species**	**Feeding Guild**	**Simplified guild**	**Species**	**Feeding Guild**	**Simplified guild**	**Species**	**Feeding Guild**	**Simplified guild**
Ixobrychus minutus	H	Insectivore	*Prunella modularis*	C	Granivore	*Parus ater*	E	Omnivore
Circus cyaneus	A	Raptor	*Erithacus rubecula*	B	Insectivore	*Parus caeruleus*	E	Omnivore
Accipiter nisus	A	Raptor	*Luscinia megarhynchos*	B	Insectivore	*Parus cristatus*	E	Omnivore
Falco tinnunculus	A	Raptor	*Luscinia svecica*	B	Insectivore	*Parus major*	E	Omnivore
Falco columbarius	A	Raptor	*Phoenicurus ochruros*	D	Insectivore	*Parus montanus*	E	Omnivore
Rallus aquaticus	H	Insectivore	*Phoenicurus phoenicurus*	E	Insectivore	*Sitta europaea*	L	Omnivore
Gallinula chloropus	H	Insectivore	*Saxicola rubetra*	G	Insectivore	*Tichodroma muraria*	E	Insectivore
Gallinago gallinago	H	Insectivore	*Oenanthe oenanthe*	D	Insectivore	*Certhia familiaris*	E	Insectivore
Scolopax rusticola	H	Insectivore	*Turdus torquatus*	B	Omnivore	*Lanius collurio*	F	Insectivore
Columba palumbus	J	Vegetarian	*Turdus merula*	B	Omnivore	*Garrulus glandarius*	L	Omnivore
Otus scops	A	Raptor	*Turdus philomelos*	B	Omnivore	*Nucifraga caryocatactes*	L	Omnivore
Glaucidium passerinum	A	Raptor	*Turdus iliacus*	B	Omnivore	*Fringilla coelebs*	C	Granivore
Asio otus	A	Raptor	*Turdus viscivorus*	B	Omnivore	*Fringilla montifringilla*	C	Granivore
Aegolius funereus	F	Insectivore	*Locustella naevia*	G	Insectivore	*Serinus serinus*	C	Granivore
Caprimulgus europaeus	E	Insectivore	*Acrocephalus scirpaceus*	G	Insectivore	*Serinus citrinella*	C	Granivore
Jinx torquilla	E	Insectivore	*Hippolais icterina*	E	Omnivore	*Carduelis carduelis*	C	Granivore
Picus viridis	E	Insectivore	*Sylvia melanocephala*	E	Omnivore	*Carduelis spinus*	C	Granivore
Dryocopus martius	E	Insectivore	*Sylvia curruca*	E	Omnivore	*Carduelis cannabina*	C	Granivore
Dendrocopos major	E	Insectivore	*Sylvia communis*	G	Omnivore	*Carduelis flammea*	C	Granivore
Lullula arborea	C	Granivore	*Sylvia borin*	E	Omnivore	*Loxia curvirostra*	C	Granivore
Alauda arvensis	C	Granivore	*Sylvia atricapilla*	E	Omnivore	*Pyrrhula pyrrhula*	C	Granivore
Hirundo rustica	F	Insectivore	*Phylloscopus sibilatrix*	E	Omnivore	*Coccothraustes coccothra*	C	Granivore
Delichon urbica	F	Insectivore	*Phylloscopus collybita*	E	Omnivore	*Emberiza cirlus*	C	Granivore
Anthus trivialis	D	Insectivore	*Phylloscopus trochilus*	E	Omnivore	*Emberiza citrinella*	C	Granivore
Anthus pratensis	D	Insectivore	*Regulus regulus*	E	Insectivore	*Embriza cia*	C	Granivore
Anthus spinoletta	D	Insectivore	*Regulus ignicapillus*	E	Insectivore	*Emberiza hortulana*	C	Granivore
Motacilla flava	D	Insectivore	*Muscicapa striata*	F	Insectivore	*Emberiza schoeniclus*	C	Granivore
Motacilla alba	D	Insectivore	*Ficedula hypoleuca*	F	Omnivore			
Troglodytes troglodytes	L	Omnivore	*Aegithalos caudatus*	E	Omnivore			
